# Histopathology of the tongue in a hamster model of COVID-19

**DOI:** 10.1186/s12903-025-05420-9

**Published:** 2025-01-23

**Authors:** John Coggins, Marina Hosotani Saito, Rebecca Cook, Shinji Urata, Megumi Urata, Nantian Lin Harsell, Wilhelmina Nanrui Tan, Bibiana Toro Figueira, Megan Bradley, Nadia Z. Quadri, Janisah Amirah I. Saripada, Rachel A. Reyna, Junki Maruyama, Slobodan Paessler, Tomoko Makishima

**Affiliations:** 1 Department of Otolaryngology, University of Texas Medical Branch at Galveston, Galveston, TX USA; 2 School of Medicine, University of Texas Medical Branch, Galveston, TX USA; 3 Department of Pathology, University of Texas Medical Branch, Galveston, TX USA

**Keywords:** SARS-CoV-2, Dysgeusia, Vallate papillae

## Abstract

**Objective:**

With altered sense of taste being a common symptom of coronavirus disease 2019 (COVID-19), the main objective was to investigate the presence and distribution of severe acute respiratory syndrome coronavirus-2 (SARS-CoV-2) within the tongue over the course of infection.

**Methods:**

Golden Syrian hamsters were inoculated intranasally with SARS-CoV-2 and tongues were collected at 2, 3, 5, 8, 17, 21, 35, and 42 days post-infection (dpi) for analysis. In order to test for gross changes in the tongue, the papillae of the tongue were counted. Paraffin-embedded thin sections of the tongues were labeled for the presence of SARS-CoV-2 antigen.

**Results:**

There was no difference in fungiform or filiform papillae density throughout the course of infection. SARS-CoV-2 antigen was observed in the vallate papillae taste buds (3–35 dpi) and autonomic ganglia (5–35 dpi), as well as in the serous and mucous salivary glands of the posterior tongue (2–42 dpi).

**Conclusion:**

The presence and distribution of SARS-CoV-2 suggest that the virus could cause taste disturbance by infecting the vallate papillae taste buds. This effect could be exacerbated by a diminished secretion of saliva caused by infection of the serous salivary glands and the autonomic ganglia which innervate them.

**Supplementary Information:**

The online version contains supplementary material available at 10.1186/s12903-025-05420-9.

## Background

Severe acute respiratory syndrome coronavirus-2 (SARS-CoV-2) infection can cause anosmia and ageusia with sizeable prevalence rates of 38.2% and 36.6%, respectively [1, 2]. These symptoms have played significant roles in the epidemiology of the coronavirus disease 2019 (COVID-19) pandemic as they are early manifestations of the disease and allow the infected to be identified before traditional viral respiratory symptoms are noted [3–5]. In some cases, they may be the sole manifestations of COVID-19, further enhancing the ability to contact trace and quickly quarantine those infected [6, 7]. In light of this, the Centers for Disease Control and Prevention added loss of taste and smell to the list of COVID-19 symptoms in April of 2020. While anosmia and ageusia serve significant epidemiological purposes, they are equally significant in their affront on quality of life. The loss of taste and smell can be distressing and is associated with increased rates of depression and suicidal ideation [8]. The median duration for COVID-19 related anosmia and dysgeusia is seven days; however, some individuals report these symptoms for significantly greater periods of time [9]. Moreover, vaccination has been shown to not prevent or mitigate the onset of anosmia [10].

**Fig. 1:**
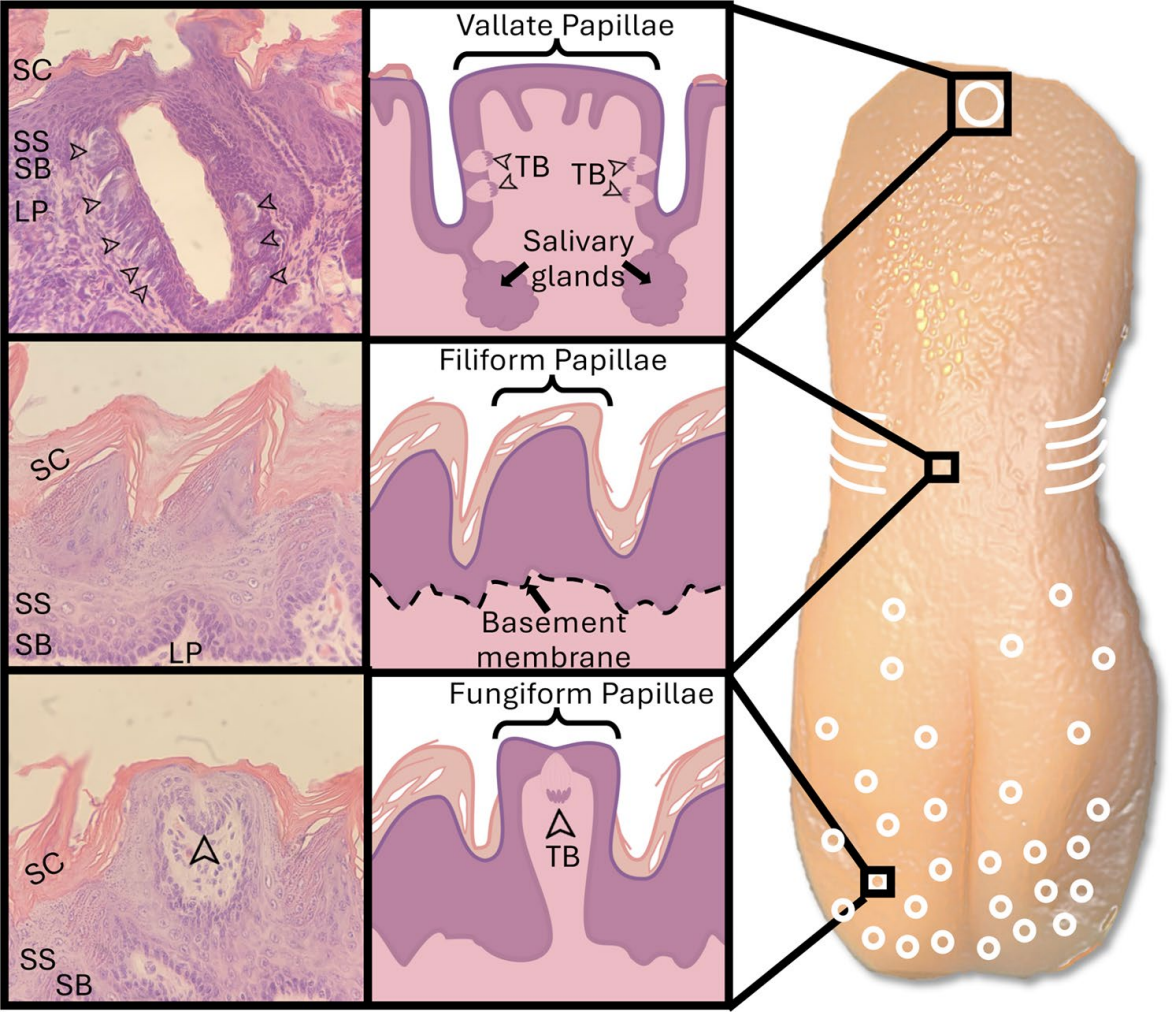
Anatomy of the hamster tongue. Distribution of tongue papillae and related histology. A midline vallate papillae resides in the posterior region (white oval), foliate papillae on the sides (white curved lines), fungiform papillae on the anterior (white circles), and filiform papillae filling the remaining space. Taste buds (arrowheads) reside on the sides of the vallate papillae and the tips of the fungiform papillae but are not found on the filiform papillae. Salivary glands are located beneath the troughs of vallate papillae and help dissolve food molecules. SC; stratum cornuem, SS; stratum spinosum, SB; stratum basale, LP; lamina propria, TB; taste buds

The sensation of taste is dependent upon multiple structures and processes. Initially, salivary glands produce saliva to dissolve tastants [11]. The parotid, submandibular, and sublingual salivary glands produce the majority of saliva but are supplemented by the serous salivary glands of von Ebner, which are located directly underneath the furrows of the vallate papillae (Fig. 1) [11]. Once dissolved, food molecules enter taste buds through taste pores and bind to their respective taste cell receptors. A signal cascade converts the chemical signal to a neural impulse, which then travels to the brain [11]. These signals are heavily supplemented by the sense of smell, which is often purported to contribute 75–95% of the perceived sense of taste; however, given the difficulty in quantifying perceived taste sensation, these numbers have undergone little empirical validation [12]. The loss of function of any of these structures is capable of causing taste disturbance [11]. In fact, ageusia boasts a wide variety of etiologies, including drug-induced, age-related, neuropathic, dietary deficiency, systemic condition, and iatrogenic (radiation, chemotherapy, or surgery), and the pathophysiology for each varies according to its etiology [13]. For example, the loss of saliva in Sjögren’s syndrome disrupts the dissolution of food molecules and decreases taste sensation, while zinc deficiency causes ageusia by limiting the production of important salivary enzymes [13, 14].

SARS-CoV-2 may cause ageusia through a variety of processes. One hypothesis postulates that SARS-CoV-2 induces ageusia by activating toll-like receptors and interferon receptors in the taste buds, stimulating inflammatory processes and impeding taste cell regeneration [15]. A separate hypothesis suggests that SARS-CoV-2 could have an effect on the nervous system [16]. Studies have shown that SARS-CoV-2 is capable of using the trigeminal and olfactory nerves to travel into the central nervous system. Therefore, COVID-19 may cause ageusia by eliciting T cell-mediated autoimmune damage and demyelination while traveling through nerves [16]. Finally, the angiotensin converting enzyme 2 (ACE2) receptor has been implicated in the pathophysiology of taste dysfunction [17]. Taste dysfunction is a rare side effect of ACE inhibitor therapy, and SARS-CoV-2 downregulation of ACE2 may cause dysgeusia through a similar mechanism [18, 19]. A better understanding of the taste structures that are affected by the SARS-CoV-2 virus could help explain the pathophysiology of SARS-CoV-2 related taste dysfunction. This study aims to reduce this gap in knowledge by determining the location of the SARS-CoV-2 virus within the tongue over the course of an infection in hamsters.

## Materials and methods

### Animal experiment

A hamster model was chosen for this study because golden Syrian hamsters are susceptible to direct clinical isolates of SARS-CoV-2 virus without the need of genetic modifications [20–22], the hamsters are immunocompetent and feature low mortality with significant lung pathology, and they are previously well characterized for olfactory studies [23, 24]. Additionally, hamster models have been historically utilized to study taste physiology [25]. The hamster tongues used in this study were derived from a study on anosmia after SARS-CoV-2 infection; details are published in the previous report [24]. Briefly, five to six-week-old female Golden Syrian hamsters were inoculated intranasally with 100 µL of 10^5^ median tissue culture infectious dose of SARS-CoV-2 alpha-strain diluted with phosphate-buffered saline or a vehicle control. The infected hamsters were divided into 8 groups (*n* = 4 each) with tongues harvested at 2, 3, 5, 8, 17, 21, 35, and 42 days post-infection (dpi). In this study chemosensory deficit in olfaction was confirmed in infected hamsters by measuring the duration of time required to find a buried cookie. The hamsters showed significant anosmia at days 2, 3, and 5 dpi that progressively recovered through 42 dpi as shown by the food detection test [24]. A high-flow rate of CO_2_ followed by thoracotomy were used for euthanasia. All animal studies were reviewed and approved by the Institutional Animal Care and Use Committee at UTMB and were conducted according to the National Institutes of Health guidelines.

### Tissue processing

The whole tongue was dissected and fixed in 10% formalin for more than seven days before taken out of the biosafety level 3 laboratory per institutional regulations. The fixation time was not uniform due to unstable lab operations during the COVID-19 pandemic. The tongue was cut in the center sagittally and each half embedded in paraffin. Tissue was dehydrated in ethanol, cleared in xylene, and embedded in paraffin in the standard fashion. Tongues were then sectioned with sagittal cuts at a thickness of 5 μm. Slides were stained with hematoxylin and eosin (H&E) or labeled with SARS Nucleocapsid protein antibody (Novus Cat# NB100-56576, RRID: AB_838838).

### Papillae counting

**Fig. 2:**
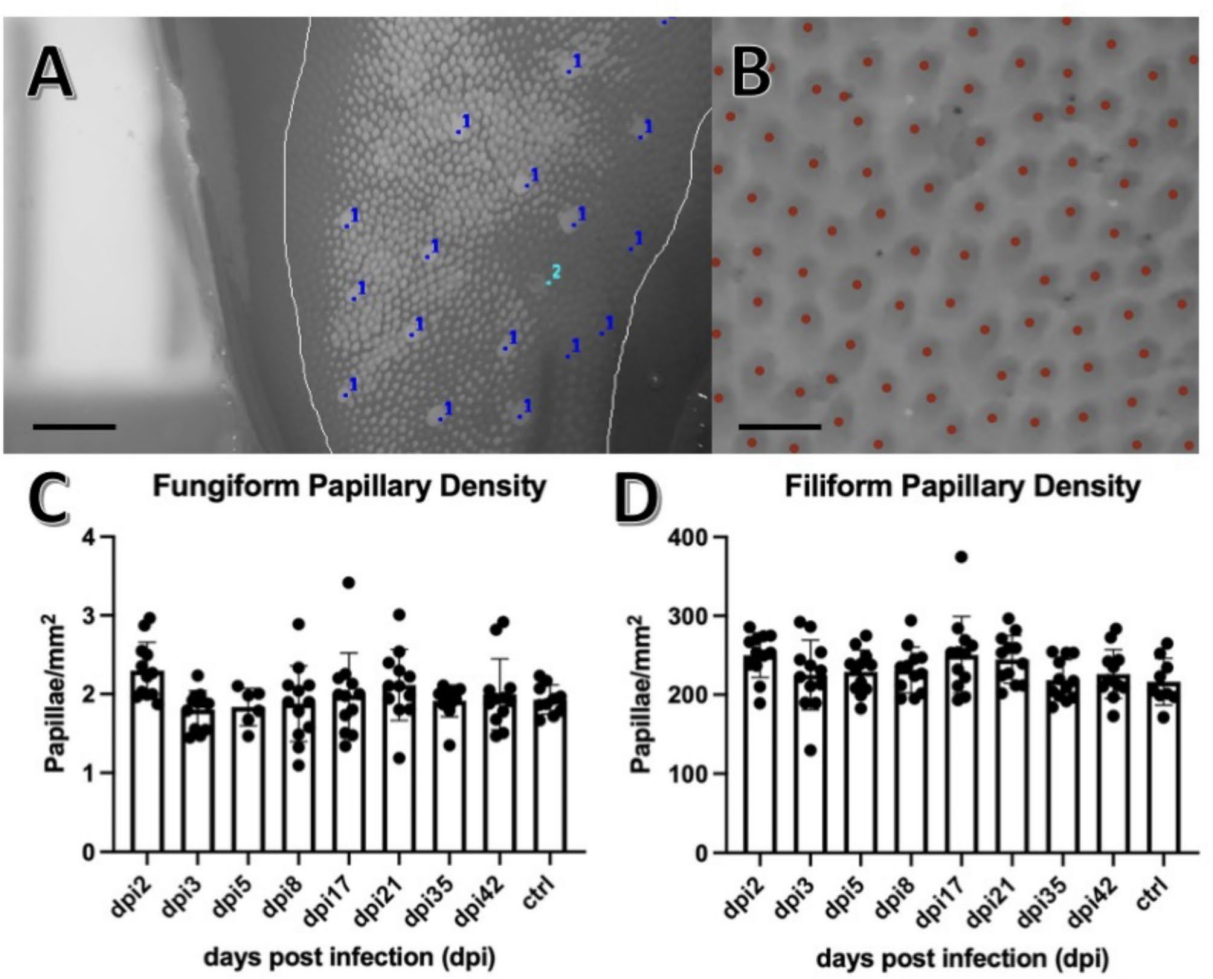
Papillae counting. (**A**) Magnified 8-bit image of the anterior tongue using Image J v1.53a. The region of interest (ROI) was demarcated (grey line) using Image J freehand tool, the fungiform papillae were counted using the Cell Counter plugin for Image J (dark blue dots), and the area (mm^2^) calculated using the measurement tool in Image J with the ruler behind the tongue for reference. Papillae were excluded from the count (light blue dot) if they were found to not be a complete circle, not completely within the ROI, or not approximately 3:1 in size when compared with surrounding filiform papillae. Scale bar indicates 500 μm. (**B**) Magnified 8-bit inverted image of the anterior tongue using Image J v1.53a. Red dots indicate computer generated counting of the papillae using the Image-based Tool for Counting Nuclei plugin for Image J. Scale bar indicates 100 μm. (**C**) Fungiform papillae densities were plotted against days post infection (dpi). Error bars indicate mean and standard deviation. No significant differences were found between any of the infected groups (dpi#) or mock (ctrl) when tested with ANOVA set to *p* < 0.05. For all groups, n = triplicates of 4. (**D**) Filiform papillae densities were plotted against dpi. Error bars indicate mean and standard deviation. No significant differences were found between any of the infected groups (dpi#) or ctrl when tested with ANOVA set to *p* < 0.05. For all groups, n = triplicates of 4

Formalin fixed hamster tongues were dyed with green food coloring (Betty Crocker Classic Gel Food Color) diluted in phosphate buffered saline to facilitate visualization of the papillae (Supp. Figure 1) [26]. Images of the anterior, middle, and posterior sections of each tongue were captured using a dissecting microscope at 25x magnification (Fig. 2). Images were converted to 8-bit black and white. Using a ruler placed in the background of the image, the scale of the image in millimeters was determined using the set scale function in Image J v1.53a. The portion of the tongue within focus was demarcated as the region of interest (ROI) using the freehand tool, and the area was measured using the measure function in Image J. Fungiform papillae within the ROI were then individually counted using the Cell Counter plugin for Image J v1.53a. Fungiform papillae were counted if they met the following criteria: the papillae were complete circles, larger than filiform papillae in an approximate ratio of 3:1, and located completely within the selected ROI. Fungiform papillae were counted within three distinct ROI using the image of the anterior tongue to create a triplicate measurement. The number of papillae and area within each section was added, and the density was calculated. Filiform papillae were counted using one ROI within each of the three sections of the tongue (anterior, middle, and posterior) using the automated counter Image-based Tool for Counting Nuclei plugin for Image J. The estimated width of each papilla was set to 30 pixels, and the minimum distance between papillae was set to 15 pixels. The automated counter was set to detect light peaks within the selected ROI. This process was repeated twice more to produce triplicate measurements for each tongue.

### Immunohistochemistry (IHC)

Thin-sectioned tongue tissue was deparaffinized, rehydrated, and subjected to heat-induced antigen retrieval in Target Retrieval Solution (S-1700, Dako). The slides were incubated with SARS Nucleocapsid Protein antibody (1:200 dilution for Supp. Figure 2 and Supp. Tables 2, 1:1,000 dilution for Supp. Figure 3 and Supp. Table 3) (Novus Biologicals Cat# NB100-56576, RRID: AB_838838) followed by incubation with a biotinylated secondary antibody (SignalStain Boost IHC Detection Reagent HRP Rabbit, Cell Signaling Technology). Lastly, slides were developed with 3,3’-Diaminobenzidine (ImmPACT DAB Substrate, Peroxidase HRP, Vector Laboratories) and counterstained with hematoxylin. In addition, four slides were stained without primary antibody in order to test non-specific binding of the secondary antibody. All four slides from 5 to 17 dpi, and mock which included all structures of interest did not show any non-specific false positive staining in the tongue.

**Fig. 3:**
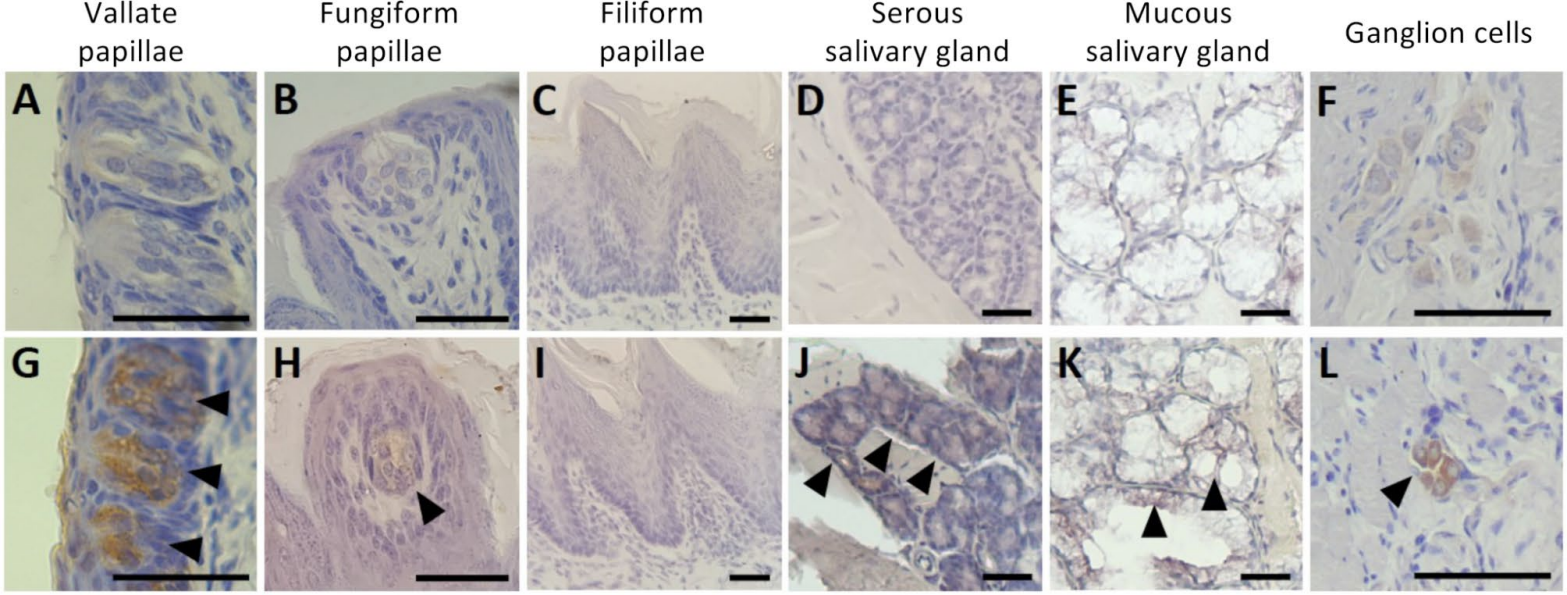
SARS-CoV-2 antigen labeling in the tongue. Representative images of immunohistochemical labeling (1:200 antibody dilution) of structures of interest with or without infection with SARS-CoV-2. The infected samples at 5 days post infection (dpi) are shown in the lower panels (**G**, **H**, **I**, **J**, **K**, and **L**), with arrowheads pointing to positive brown-colored labeling of the SARS-CoV-2 antigen, compared with negative labeling in the mock infected control samples in the upper panels (**A**, **B**, **C**, **D**, and **E**). The vallate papillae taste buds (**G**) and ganglion cells (**L**) show positive labeling, while fungiform papillae taste bud (**H**), serous salivary gland cells (**J**), and mucous salivary gland cells (**K**) show weakly positive labeling. The filiform papilla cells (**I**) were negative for labeling. Scale bars indicate 100 μm

### Histopathology grading

H&E-stained and SARS-CoV-2 antibody-labeled slides were graded by at least three independent observers blinded to the specimen information. Nine structures within the tongue were evaluated: the autonomic ganglia, serous salivary glands, mucous salivary glands, fungiform papillae, filiform papillae, vallate papillae, muscle, nerves, and vasculature. Graders evaluated H&E slides for de-epithelialization, taste bud disengagement, fibroblast cell infiltrate, and blood cell infiltrate within each of the eight structures (Supp. Figure 4). The number of cells within the single largest vallate papillae taste bud, fungiform papillae taste bud, and autonomic ganglia was recorded. Cells with visible nuclei within the structure were counted. Structures labeled with SARS-CoV-2 antibody were graded for the intensity of cell labeling on a scale of 0–2, with 0 indicating no staining, 1 mild staining, and 2 strong staining (Supp. Fig. 5 and Supp. Tables 2 and 3). If a structure was not identified within a tongue, the structure was not graded and did not contribute to the overall score (Supp. Table 1).

### Statistics

Papillary densities were compared among different timepoints and animals with a one-way ANOVA and post hoc Tukey test in Prism v9 with *p* < 0.05 determining a statistically significant difference. Scores for SARS-CoV-2 antibody labeling (Supp. Tables 2 and 3) from each dpi and mock infected group were compared within each structure of fungiform papillae taste buds, vallate papillae taste buds, serous salivary glands, mucous salivary glands, and autonomic ganglia. Four reviewers scored in Experiment 1 (1:200 dilution antibody, Supp. Table 2) and three reviewers in Experiment 2 (1:1000 dilution antibody, Supp. Table 3). The means of the reviewer scores for each of the timepoints and mock group were compared. Since the data did not follow normality assumption and the sample size was small, non-parametric Kruskal-Wallis test was performed for median score comparison (*p* < 0.05, significant difference). And for pairwise comparison between each of the timepoints, Dwass, Steel, Critchlow-Fligner multiple comparison analysis was performed. The analyses were performed with SAS version 9.4 (SAS, Inc., Cary, NC).

## Results

Counting of the fungiform and filiform papillae indicated there was no change in the number of papillae throughout the course of SARS-CoV-2 infection (Fig. 2). The staining results (H&E) revealed no de-epithelialization, taste bud disengagement, or cellular infiltrate at any time point from 2 to 42 dpi. The numbers of cells within the infected vallate papillae taste buds (6.4 ± 2.9), fungiform papillae taste buds (7.9 ± 1.8), and autonomic ganglia (6.3 ± 1.7), were not statistically different from the number of cells within the same structures of control tongues (vallate papillae 8.0 ± 1, fungiform papillae 6.8 ± 0.7, autonomic ganglia 7.8 ± 1.2) (mean ± standard deviation).

IHC of the hamster tongues revealed the presence of SARS-CoV-2 antigen post-infection in the cytoplasm of cells in the vallate papillae, autonomic ganglia, and salivary glands (Fig. 3, Supp. Figures 2 and 3). The autonomic ganglia were labeled positively for the SARS-CoV-2 antigen at 5 through 21 dpi. At 42 dpi, the labeling decreased in the intensity and proportion of cells labeled. The vallate papillae taste buds were labeled for SARS-CoV-2 antigen at 2 through 42 dpi, and no strong positive labeling was found in the control hamsters. Serous and mucous salivary glands labeled positive at 2 to 42 dpi. The fungiform papillae taste buds showed weak labeling, most notably at 5 dpi; however, this labeling was considerably less than the labeling in the vallate papillae taste buds (Fig. 3G, H). However, SARS-CoV-2 antibody labeling scores did not show any significant difference at any timepoint after infection or when compared with mock control in any of the anatomical structures (*p* > 0.05) (Supp. Tables 2 and 3). SARS-CoV-2 antigen was not detected in the filiform papillae (Fig. 3I), muscle, or vasculature within any samples of the infected or control groups.

## Discussion

Fungiform papillae density is implicated as a proxy for taste sensitivity, with higher densities indicating greater taste sensation [27], although some research has brought this into question [28, 29]. Fungiform papillae density is not often used as a metric for acute injury or infective agent is unlikely to alter the number of papillae. For this reason, a change in papillae density was not expected. Indeed, papillae densities in the hamster tongues of the present study were similar to that at baseline with comparable numbers of papillae across infected and control groups. This experiment provides valuable documentation that the number of fungiform papillae did not decrease as a result of infection.

IHC revealed the presence of SARS-CoV-2 antigen in three structures: the vallate papillae taste buds, autonomic ganglia, and the salivary glands. A viral infection in any of these structures could affect the sense of taste by directly disrupting cell function in the taste buds or by decreasing saliva production [30, 31], as xerostomia is a recognized symptom of the infection [32]. The vallate papillae house approximately half of all taste buds in the human tongue [33], and the present experiments consistently detected SARS-CoV-2 antigen in these taste buds, indicating SARS-CoV-2 infection. The mechanism by which SARS-CoV-2 may damage taste cells is unknown, but one possible pathway includes inflammatory cytokines and toll-like receptors [14]. Autonomic ganglia adjacent to nerves within the tongue controls the secretion of saliva from glands within the tongue [34]. An infection of the autonomic ganglia could reduce saliva production and prevent tastants from dissolving and binding to taste cell receptors. SARS-CoV-2 antigen was detected in the salivary glands throughout the study from 2 to 42 dpi, suggesting a potential mechanism for taste loss via decreased saliva.

The COVID-19 hamster model showed deterioration of olfactory function mainly in the early days starting from 2 dpi to at least 5 dpi after infection with SARS-CoV-2 virus alpha strain [24]. In comparison, the labeling with the SARS-CoV-2 nucleocapsid antibody in the tongue was observed in a delayed timeline with vallate papillae taste buds starting from 3 dpi and the autonomic ganglia showing the strongest labeling at 5–17 dpi. It is speculated that this difference is due to the accessibility of the virus to the target cells. The surface of the tongue is covered with thick keratin, which may protect the virus from penetrating into deeper cell layers initially after infection. However, over time, the virus particles that may have migrated through the openings of the salivary glands may be able to spread into deeper structures. The short-lived taste disturbance may be caused by direct changes to the cells with a faster turnover, such as the taste cells and the salivary gland cells, interfering with the delivery of the taste molecules. Conversely, the prolonged taste disturbance may be determined by the extent of viral penetration into cells with slower or no turnover, such as the autonomic ganglia.

The primary means of entry into the cell for SARS-CoV-2 is the ACE2 receptor [35], which is expressed in the filiform papillae, type 2 taste cells, and autonomic ganglia cell bodies [36–38]. In this study, SARS-CoV-2 antigen was not detected in the filiform papillae. This result could be related to the thick keratin layer that may protect the filiform papillae from viral attack. It is important to note that the filiform papillae, lacking taste buds, do not contribute to the sense of taste, and an infection of these structures would not affect the ability to taste. Type 2 taste cells contain G protein-coupled receptors (GPCRs) for either bitter, umami, or sweet tastants [39]. These cells are found primarily within the taste buds of the fungiform and vallate papillae in humans, whose combined taste buds account for most of the taste sensation [11]. In hamsters, vallate papillae contribute 23%, and foliate and fungiform papillae contribute 32% and 18%, respectively [40]. Due to the orientation of sectioning we did not evaluate the foliate papillae histology. In this study, the fungiform papillae taste buds had minimal SARS-CoV-2 antigen labeling, while vallate papillae taste buds showed marked labeling from 3 to 35 dpi. The infection of these taste buds likely coincides with the ACE2 expression within the type 2 taste cells, leading to taste disturbance [37]. Similar findings in human studies provide evidence for a mechanism where SARS-CoV-2 directly infects type 2 taste cells via ACE2 receptors, causing taste bud dagame, inflammation, and neurite damage [41–43].

The length of infection in the tongue is speculated to be crucial for the prolonged taste disturbance. In this hamster model, SARS-CoV-2 cleared from the lungs by 8 dpi [24]. On the other hand, SARS-CoV-2 existed within the tongue through 35 dpi, suggesting long-lasting viral production within the tongue. In humans, SARS-CoV-2 viral products have been detected in tongues up to 63 days after infection, with duration of viral products within the tongue correlating to taste disturbance [41]. Typically, patients develop chemosensory changes 4–5 days after the onset of other symptoms and recover their sense of taste and smell 7–14 days later [42]. This time course is consistent with the findings in this study, as minimal SARS-CoV-2 antigen labeling was noted at 2 dpi which quickly increases thereafter and persisting through 35 dpi, which is beyond the estimated recovery of taste loss. This discrepancy could occur due to a regain of taste bud function despite a continued presence of viral antigens. Atypically, patients experience prolonged or permanent loss of taste. It is possible that a separate mechanism produces this prolonged version of COVID-19 related taste dysfunction. Conversely, this study’s sample size may have been too small to capture the particular type of infection leading to prolonged taste disturbance.

The identification of the peak timepoint of infection was failed, as SARS-CoV-2 antibody labeling scores showed no statistically significant differences among any of the experimental groups, in both Experiment 1 (SARS-CoV-2 antibody 1:200 dilution, Supp. Table 2) and in Experiment 2 (SARS-CoV-2 antibody 1:1000 dilution, Supp. Table 3). However, due to the overall small sample size and loss of datapoints (Supp. Table 1), quantification of the dataset and the results should be interpreted with caution. The statistical significance may also be affected by the incomplete penetrance of SARS-CoV-2 related taste disturbance, in which case infected hamsters may not display positive antibody staining within their tongues.

This study indicated that the pathophysiology of SARS-CoV-2-induced taste dysfunction likely involves direct infection of taste cells coupled with decreased saliva production. Therefore, therapies which prevent taste cell damage and treat xerostomia would improve taste sensation. Theophylline may improve taste cell function by augmenting GPCRs signaling in taste buds [44]. Secretagogues like cevimeline and pilocarpine could treat xerostomia [45]. Lastly, zinc supplementation may be beneficial, as hypozincemia has been linked to both taste dysfunction and xerostomia in SARS-CoV-2 infection [46]. Further research is needed to elucidate the underlying mechanisms and develop effective treatments against SARS-CoV-2 infection-induced taste dysfunction.

## Conclusion

SARS-CoV-2 antigen was detected in the vallate papillae taste buds, salivary glands, and autonomic ganglia of the tongue of the hamster model of COVID-19. The present results suggest that the loss of function of the vallate papillae taste buds, salivary glands, or autonomic ganglia may individually or in combination result in COVID-19-related taste disturbance.

## Electronic supplementary material

Below is the link to the electronic supplementary material.

Supplementary Material 1

Supplementary Material 2

## Data Availability

All data generated and analysed in this study are included in this published article and its supplementary figures and tables.

## Abbreviations

ACE2: Angiotensin converting enzyme 2
COVID 19: Coronavirus disease 2019
dpi: Days post infection
H&E: Hematoxylin and eosin
IHC: Immunohistochemistry
ROI: Region of interest
SARS-CoV-2: Severe acute respiratory syndrome coronavirus 2
