# Supplementary material for: Histopathology of the tongue in a hamster model of COVID-19
Source: BMC Oral Health. 2025 Jan 23;25:121. doi: 10.1186/s12903-025-05420-9 (PMC11755867; doi:10.1186/s12903-025-05420-9)
Supplement: Supplementary file 1 — Supplementary Material 1 [file 12903_2025_5420_MOESM1_ESM.docx]

**Supplementary Table 1: Presence/absence of anatomical structures in tongue thin section slides for each sample.**

| dpi | Sample No. | Taste buds | | | | | | Salivary gland cells | | | | | | Ganglion cells | | |
| --- | --- | --- | --- | --- | --- | --- | --- | --- | --- | --- | --- | --- | --- | --- | --- | --- |
|  |  | in fungiform papillae | | | in vallate papillae | | | Serous | | | Mucous | | |  |  |  |
|  |  | H&E | 1:200 | 1:1000 | H&E | 1:200 | 1:1000 | H&E | 1:200 | 1:1000 | H&E | 1:200 | 1:1000 | H&E | 1:200 | 1:1000 |
| 2 | 1 | + | + | + | + | + | + | + | + | + | - | - | - | + | + | + |
|  | 2 | + | + | + | + | - | - | + | + | + | - | - | - | - | - | + |
|  | 3 | + | + | + | + | - | + | + | + | + | - | - | - | - | - | + |
|  | 4 | + | + | + | - | - | - | + | + | + | + | + | + | - | - | - |
| 3 | 5 | + | + | - | - | + | + | + | + | + | - | - | - | - | - | - |
|  | 6 | + | + | + | + | - | - | + | + | - | - | - | - | - | - | - |
|  | 7 | + | + | - | - | - | + | + | + | + | - | - | - | + | - | - |
|  | 8 | + | + | - | + | - | - | + | + | + | - | - | - | + | - | - |
| 5 | 9 | - | - | + | - | - | - | + | + | - | - | - | - | + | - | - |
|  | 10 | + | + | + | - | - | + | + | + | + | - | - | - | - | - | - |
|  | 11 | + | + | + | + | + | - | + | + | + | - | - | - | + | - | + |
|  | 12 | - | + | + | + | + | - | + | + | + | + | + | + | - | - | + |
| 8 | 13 | + | + | + | - | - | + | + | + | + | - | - | - | - | - | - |
|  | 14 | - | + | - | - | - | + | + | - | + | - | - | - | + | + | - |
|  | 15 | + | + | + | + | + | + | + | + | + | + | + | + | + | + | - |
|  | 16 | - | + | + | + | + | + | + | + | + | - | - | - | - | - | + |
| 17 | 17 | + | + | + | - | - | + | + | + | + | + | + | + | - | - | + |
|  | 18 | + | + | + | - | - | + | + | + | - | - | - | - | - | + | - |
|  | 19 | + | + | + | - | - | - | + | + | - | - | - | - | + | - | - |
|  | 20 | + | + | + | - | - | + | + | + | + | - | - | - | - | + | + |
| 21 | 21 | + | + | + | - | - | - | + | + | + | - | - | - | + | + | - |
|  | 22 | + | + | - | - | - | - | + | + | + | - | - | - | + | - | - |
|  | 23 | - | + | - | - | - | - | + | + | - | - | - | - | - | - | - |
|  | 24 | + | + | + | - | - | + | + | + | + | - | - | - | - | + | - |
| 35 | 25 | + | + | - | - | - | - | + | + | - | - | - | - | + | + | - |
|  | 26 | + | + | + | - | - | - | + | + | + | + | + | + | + | + | + |
|  | 27 | + | + | + | + | + | - | + | + | + | - | - | - | + | - | + |
|  | 28 | - | - | - | - | - | + | + | + | + | + | + | + | - | - | + |
| 42 | 29 | - | + | - | + | + | - | + | + | - | - | - | - | - | - | - |
|  | 30 | - | - | + | + | + | - | + | + | - | - | - | - | - | - | - |
|  | 31 | - | - | - | - | - | - | + | + | - | - | - | - | - | - | - |
|  | 32 | - | - | - | + | + | - | + | + | + | + | + | - | + | + | + |
| Mock | 33 | + | + | - | - | - | - | + | + | + | - | - | - | + | + | - |
|  | 34 | + | + | - | - | - | - | + | + | + | - | - | - | - | - | - |
|  | 35 | - | + | - | + | + | - | + | + | + | - | - | - | - | - | - |
|  | 36 | + | + | + | - | - | - | + | + | + | + | + | + | + | + | + |
| Structures identified are indicated as a “+” symbol. Structures that were not found are indicated as a “-” symbol. Information on all slides used for grading H&E and immunohistochemistry for SARS-CoV-2 antibody (1:200 and 1:1000) are listed. | | | | | | | | | | | | | | | | |

**Supplementary Table 2: Scoring of intensity of SARS-CoV-2 antibody reaction graded by 4 reviewers, immunohistochemistry experiment 1 (1:200 dilution)**.

| dpi | Sample No. | Taste buds | | | | | | | | | | Salivary gland cells | | | | | | | | | | Ganglion cells | | | | |
| --- | --- | --- | --- | --- | --- | --- | --- | --- | --- | --- | --- | --- | --- | --- | --- | --- | --- | --- | --- | --- | --- | --- | --- | --- | --- | --- |
|  |  | in fungiform papillae | | | | | in vallate papillae | | | | | Serous | | | | | Mucous | | | | |  |  |  |  |  |
|  |  | R1 | R2 | R3 | R4 | Ave | R1 | R2 | R3 | R4 | Ave | R1 | R2 | R3 | R4 | Ave | R1 | R2 | R3 | R4 | Ave | R1 | R2 | R3 | R4 | Ave |
| 2 | 1 | 0 | 0 | 1 | 0 | 0.25 | 0 | - | - | 0 | 0 | 0 | 0 | - | 0 | 0 | - | - | - | - | - | 0 | - | 0 | - | 0 |
|  | 2 | 0 | 0 | - | - | 0 | - | - | - | - | - | 0 | 0 | 0 | 0 | 0 | - | - | - | - | - | - | - | - | - | - |
|  | 3 | 0 | 0 | 0 | - | 0 | - | - | - | - | - | 0 | 0 | 0 | 0 | 0 | - | - | - | - | - | - | - | - | - | - |
|  | 4 | 0 | 0 | 0 | - | 0 | - | - | - | - | - | 0 | 0 | 0 | 0 | 0 | 0 | 0 | 0 | 0 | 0 | - | - | - | - | - |
| 3 | 5 | 0 | 0 | 0 | 0 | 0 | 2 | - | - | 2 | 2 | 0 | 0 | 0 | 0 | 0 | - | - | - | - | - | - | - | - | - | - |
|  | 6 | 0 | 0 | 0 | - | 0 | - | - | - | - | - | 0 | 0 | 0 | 0 | 0 | - | - | - | - | - | - | - | - | - | - |
|  | 7 | - | 0 | 0 | - | 0 | - | - | - | - | - | 0 | 0 | 0 | 0 | 0 | - | - | - | - | - | - | - | - | - | - |
|  | 8 | 1 | - | 1 | - | 1 | - | - | - | - | - | 0 | 0 | 1 | 0 | 0.25 | - | - | - | - | - | - | - | - | - | - |
| 5 | 9 | - | - | - | - | - | - | - | - | - | - | 0 | 0 | 0 | 0 | 0 | - | - | - | - | - | - | - | - | - | - |
|  | 10 | 0 | - | 1 | - | 0.5 | - | - | - | - | - | 0 | 0 | 0 | 0 | 0 | - | - | - | - | - | - | - | - | - | - |
|  | 11 | 1 | - | - | - | 1 | - | 0 | 0 | - | 0 | - | 0 | - | 0 | 0 | - | - | - | - | - | - | - | - | - | - |
|  | 12 | - | - | 0 | - | 0 | 1 | - | 0 | 0 | 0.33 | 0 | - | - | - | 0 | 1 | 2 | 0 | 1 | 1 | - | - | - | - | - |
| 8 | 13 | 0 | 0 | - | 0 | 0 | - | - | - | - | - | - | - | - | 0 | 0 | - | - | - | - | - | - | - | - | - | - |
|  | 14 | - | - | 0 | - | 0 | - | - | - | - | - | - | - | - | - | - | - | - | - | - | - | 2 | 2 | - | - | 2 |
|  | 15 | - | 0 | 0 | - | 0 | - | 0 | 1 | 0 | 0.33 | 1 | 1 | 2 | 0 | 1 | 1 | 1 | 2 | 2 | 1.5 | 2 | - | 2 | - | 2 |
|  | 16 | - | - | 0 | - | 0 | 2 | 2 | 0 | 2 | 1.5 | 0 | 0 | 0 | 0 | 0 | - | - | - | - | - | - | - | - | - | - |
| 17 | 17 | 0 | - | 1 | - | 0.5 | - | - | - | - | - | 0 | 0 | 0 | - | 0 | 0 | 0 | 1 | 0 | 0.25 | - | - | - | - | - |
|  | 18 | 0 | 0 | - | 0 | 0 | - | - | - | - | - | - | - | - | 0 | 0 | - | - | - | - | - | 1 | - | 1 | - | 1 |
|  | 19 | - | 0 | 0 | - | 0 | - | - | - | - | - | 0 | 0 | - | - | 0 | - | - | - | - | - | - | - | - | - | - |
|  | 20 | 0 | 0 | 0 | 0 | 0 | - | - | - | - | - | 0 | 0 | 2 | 0 | 0.5 | - | - | - | - | - | 1 | - | - | 1 | 1 |
| 21 | 21 | 0 | 0 | 0 | 0 | 0 | - | - | - | - | - | 0 | 0 | - | - | 0 | - | - | - | - | - | 1 | 1 | - | 1 | 1 |
|  | 22 | - | 0 | 0 | - | 0 | - | - | - | - | - | 0 | 0 | - | 0 | 0 | - | - | - | - | - | - | - | - | - | - |
|  | 23 | - | - | 0 | - | 0 | - | - | - | - | - | - | - | 0 | - | 0 | - | - | - | - | - | - | - | - | - | - |
|  | 24 | - | 1 | - | - | 1 | - | - | - | - | - | 0 | 0 | 0 | 0 | 0 | - | - | - | - | - | 2 | 2 | 2 | 2 | 2 |
| 35 | 25 | 0 | 1 | 0 | 1 | 0.5 | - | - | - | - | - | 0 | 0 | 1 | 0 | 0.25 | - | - | - | - | - | 0 | - | 0 | - | 0 |
|  | 26 | 0 | 1 | - | 0 | 0.33 | - | - | - | - | - | - | - | - | 0 | 0 | 1 | 2 | 0 | 1 | 1 | 1 | - | - | - | 1 |
|  | 27 | - | - | - | 0 | 0 | 2 | - | - | - | 2 | 0 | 1 | - | 0 | 0.33 | - | - | - | - | - | - | - | - | - | - |
|  | 28 | - | - | - | - | - | - | - | - | - | - | 1 | 0 | 0 | 0 | 0.25 | 0 | 0 | 0 | 0 | 0 | - | - | - | - | - |
| 42 | 29 | - | - | 0 | - | 0 | 2 | - | - | 2 | 2 | 0 | 0 | 0 | 0 | 0 | - | - | - | 0 | 0 | - | - | - | - | - |
|  | 30 | - | - | - | - | - | 1 | 0 | - | 0 | 0.33 | 0 | 0 | 0 | 0 | 0 | - | - | - | - | - | - | - | - | - | - |
|  | 31 | - | - | - | - | - | - | - | - | - | - | - | 0 | - | 0 | 0 | - | - | - | - | - | - | - | - | - | - |
|  | 32 | - | - | - | - | - | 0 | - | - | 0 | 0 | 0 | - | 0 | 0 | 0 | 0 | - | 1 | 0 | 0.33 | 1 | - | 1 | 1 | 1 |
| Mock | 33 | 0 | 1 | 1 | - | 0.67 | - | - | - | - | - | 0 | 0 | - | - | 0 | - | - | - | - | - | 0 | 0 | 0 | 0 | 0 |
|  | 34 | 0 | 0 | 0 | 0 | 0 | - | - | - | - | - | 0 | 0 | 0 | 0 | 0 | - | - | - | - | - | - | - | - | - | - |
|  | 35 | - | 0 | - | - | 0 | 0 | 0 | - | 1 | 0.33 | 0 | 0 | 1 | 0 | 0.25 | - | - | - | - | - | - | - | - | - | - |
|  | 36 | 0 | 0 | 0 | 1 | 0.25 | - | - | - | - | - | 0 | 0 | 0 | 0 | 0 | 0 | 0 | 0 | 0 | 0 | 1 | 1 | 0 | 0 | 0.5 |
| Nonparametric Kruskal Wallis test, *p*-value | |  |  |  |  | 0.64 |  |  |  |  | 0.43 |  |  |  |  | 0.67 |  |  |  |  | 0.51 |  |  |  |  | 0.17 |
| The taste buds within the fungiform papillae, vallate papillae, serous salivary glands, mucous salivary glands, and autonomic ganglia were graded on a scale of 0-2 for the intensity of SARS-CoV-2 labeling. There was no significant difference in scores among any timepoint in any structure (*p* > 0.05). dpi; days post-infection, R1-4; reviewer 1-4. | | | | | | | | | | | | | | | | | | | | | | | | | | |

**Supplementary Table 3: Scoring of intensity of SARS-CoV-2 antibody reaction graded by 3 reviewers, immunohistochemistry experiment 2 (1:1000 dilution)**.

| dpi | Sample No. | Taste buds | | | | | | | | Salivary gland cells | | | | | | | | Ganglion cells | | | |
| --- | --- | --- | --- | --- | --- | --- | --- | --- | --- | --- | --- | --- | --- | --- | --- | --- | --- | --- | --- | --- | --- |
|  |  | in fungiform papillae | | | | in vallate papillae | | | | Serous | | | | Mucous | | | |  |  |  |  |
|  |  | R1 | R2 | R3 | Ave | R1 | R2 | R3 | Ave | R1 | R2 | R3 | Ave | R1 | R2 | R3 | Ave | R1 | R2 | R3 | Ave |
| 2 | 1 | 0 | 0 | - | 0 | - | 0 | - | 0 | 0 | 0 | 0 | 0 | - | - | - | - | 0 | - | 0 | 0 |
|  | 2 | 0 | 0 | - | 0 | - | - | - | - | - | 0 | - | 0 | - | - | - | - | 1 | 0 | 1 | 0.67 |
|  | 3 | - | 0 | - | 0 | 0 | 0 | 2 | 0.67 | 0 | 1 | 1 | 0.67 | - | - | - | - | 1 | - | 0 | 0.5 |
|  | 4 | 0 | 0 | 0 | 0 | - | - | - | - | 0 | 0 | 2 | 0.67 | 2 | 2 | 2 | 2 | - | - | - | - |
| 3 | 5 | - | - | - | - | 0 | 0 | 1 | 0.33 | 0 | 0 | 1 | 0.33 | - | - | - | - | - | - | - | - |
|  | 6 | 0 | - | 0 | 0 | - | - | - | - | - | - | - | - | - | - | - | - | - | - | - | - |
|  | 7 | - | - | - | - | 0 | - | 1 | 0.5 | 0 | 0 | 1 | 0.33 | - | - | - | - | - | - | - | - |
|  | 8 | - | - | - | - | - | - | - | - | 0 | 0 | 1 | 0.33 | - | - | - | - | - | - | - | - |
| 5 | 9 | - | 0 | 0 | 0 | - | - | - | - | - | - | - | - | - | - | - | - | - | - | - | - |
|  | 10 | - | 0 | 1 | 0.5 | 0 | - | - | 0 | 0 | 0 | 1 | 0.33 | - | - | - | - | - | - | - | - |
|  | 11 | 0 | 0 | 0 | 0 | - | - | - | - | - | 0 | - | 0 | - | - | - | - | - | 2 | - | 2 |
|  | 12 | 1 | 1 | 0 | 0.67 | - | - | - | - | 2 | 1 | 1 | 1.33 | 2 | 1 | 2 | 1.67 | 2 | - | 2 | 2 |
| 8 | 13 | 0 | 0 | - | 0 | 0 | 0 | - | 0 | 0 | 0 | - | 0 | - | - | - | - | - | - | - | - |
|  | 14 | - | - | - | - | - | - | 1 | 1 | 1 | 0 | 1 | 0.67 | - | - | - | - | - | - | - | - |
|  | 15 | - | 0 | - | 0 | - | 1 | 2 | 1.5 | 0 | 0 | 1 | 0.33 | 2 | 1 | 1 | 1.33 | - | - | - | - |
|  | 16 | 0 | 0 | 1 | 0.33 | 0 | - | - | 0 | 0 | 0 | 1 | 0.33 | - | - | - | - | - | - | 1 | 1 |
| 17 | 17 | - | - | 1 | 1 | 1 | 1 | 2 | 1.33 | 1 | 1 | 1 | 1 | 1 | 1 | 1 | 1 | 2 | - | 2 | 2 |
|  | 18 | 0 | 0 | 0 | 0 | 2 | - | - | 2 | - | - | - | - | - | - | - | - | - | - | 1 | 1 |
|  | 19 | 0 | - | - | 0 | - | - | - | - | - | - | - | - | - | - | - | - | - | - | - | - |
|  | 20 | 0 | 0 | 1 | 0.33 | 2 | 1 | 2 | 1.67 | 1 | 1 | 1 | 1 | - | - | - | - | 2 | 1 | 2 | 1.67 |
| 21 | 21 | 0 | 0 | 0 | 0 | - | - | - | - | 1 | 0 | 0 | 0.33 | - | - | - | - | - | - | - | - |
|  | 22 | - | - | - | - | - | - | - | - | 1 | 0 | 1 | 0.67 | - | - | - | - | - | - | - | - |
|  | 23 | - | - | - | - | - | - | - | - | - | - | - | - | - | - | - | - | - | - | - | - |
|  | 24 | 0 | - | 0 | 0 | 1 | 1 | 1 | 1 | 1 | 0 | 1 | 0.67 | - | - | - | - | - | - | - | - |
| 35 | 25 | - | - | - | - | - | - | - | - | - | - | - | - | - | - | - | - | - | - | - | - |
|  | 26 | 0 | 1 | 0 | 0.33 | - | - | - | - | 1 | 1 | 1 | 1 | 2 | 1 | 1 | 1.33 | 2 | 1 | 1 | 1.33 |
|  | 27 | - | - | 0 | 0 | - | - | - | - | 1 | 1 | 1 | 1 | - | - | - | - | - | - | 0 | 0 |
|  | 28 | - | - | - | - | 2 | 1 | - | 1.5 | 1 | 1 | 1 | 1 | 2 | 1 | 1 | 1.33 | 2 | 1 | 2 | 1.67 |
| 42 | 29 | - | - | - | - | - | - | - | - | - | - | - | - | - | - | - | - | - | - | - | - |
|  | 30 | - | 0 | 0 | 0 | - | - | - | - | - | - | - | - | - | - | - | - | - | - | - | - |
|  | 31 | - | - | - | - | - | - | - | - | - | - | - | - | - | - | - | - | - | - | - | - |
|  | 32 | - | - | - | - | - | - | - | - | 2 | 2 | 0 | 1.33 | 2 | - | - | 2 | 1 | - | 0 | 0.5 |
| Mock | 33 | - | - | - | - | - | - | - | - | 0 | - | - | 0 | - | - | - | - | - | - | - | - |
|  | 34 | - | - | - | - | - | - | - | - | 0 | - | - | 0 | - | - | - | - | - | - | - | - |
|  | 35 | - | - | - | - | - | - | - | - | 0 | - | - | 0 | - | - | - | - | - | - | - | - |
|  | 36 | 1 | 0 | 0 | 0.33 | - | - | - | - | 1 | 1 | 1 | 1 | 1 | 1 | 1 | 1 | 1 | - | 1 | 1 |
| Nonparametric Kruskal Wallis test, *p*-value | |  |  |  | 0.57 |  |  |  | 0.22 |  |  |  | 0.13 |  |  |  | 0.32 |  |  |  | 0.18 |
| The taste buds within the fungiform papillae, vallate papillae, serous salivary glands, mucous salivary glands, and autonomic ganglia were graded on a scale of 0-2 for the intensity of SARS-CoV-2 labeling. There was no significant difference in scores among any timepoint in any structure (*p* > 0.05). dpi; days post-infection, R1-3; reviewer 1-3. | | | | | | | | | | | | | | | | | | | | | |
