# Supplementary material for: Histopathology of the tongue in a hamster model of COVID-19
Source: BMC Oral Health. 2025 Jan 23;25:121. doi: 10.1186/s12903-025-05420-9 (PMC11755867; doi:10.1186/s12903-025-05420-9)
Supplement: Supplementary file 2 — Supplementary Material 2 [file 12903_2025_5420_MOESM2_ESM.docx]

**
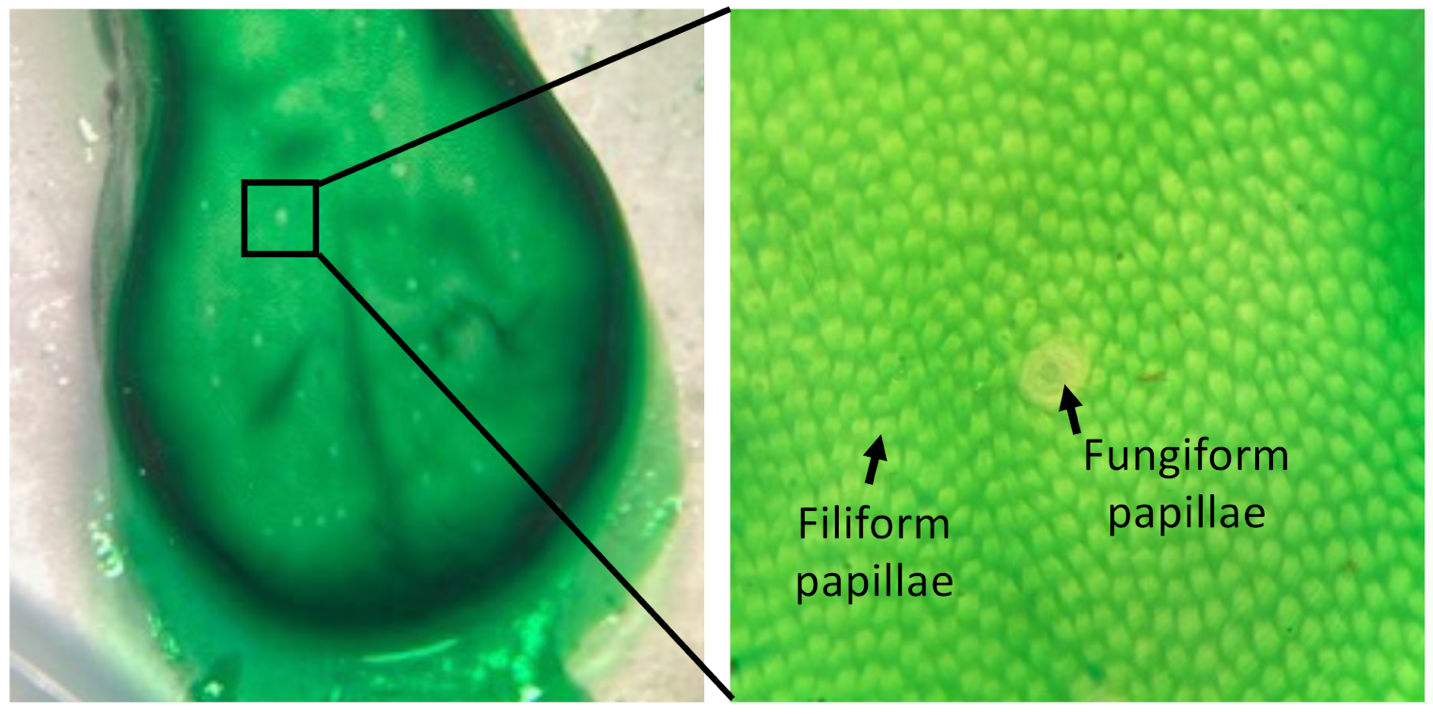
Supplemental Figure 1: Hamster tongue dyed with green food coloring**. Left image represents low magnification of the dyed tongue. The squared region was magnified into the right magnified image showing filiform and fungiform papillae.

**
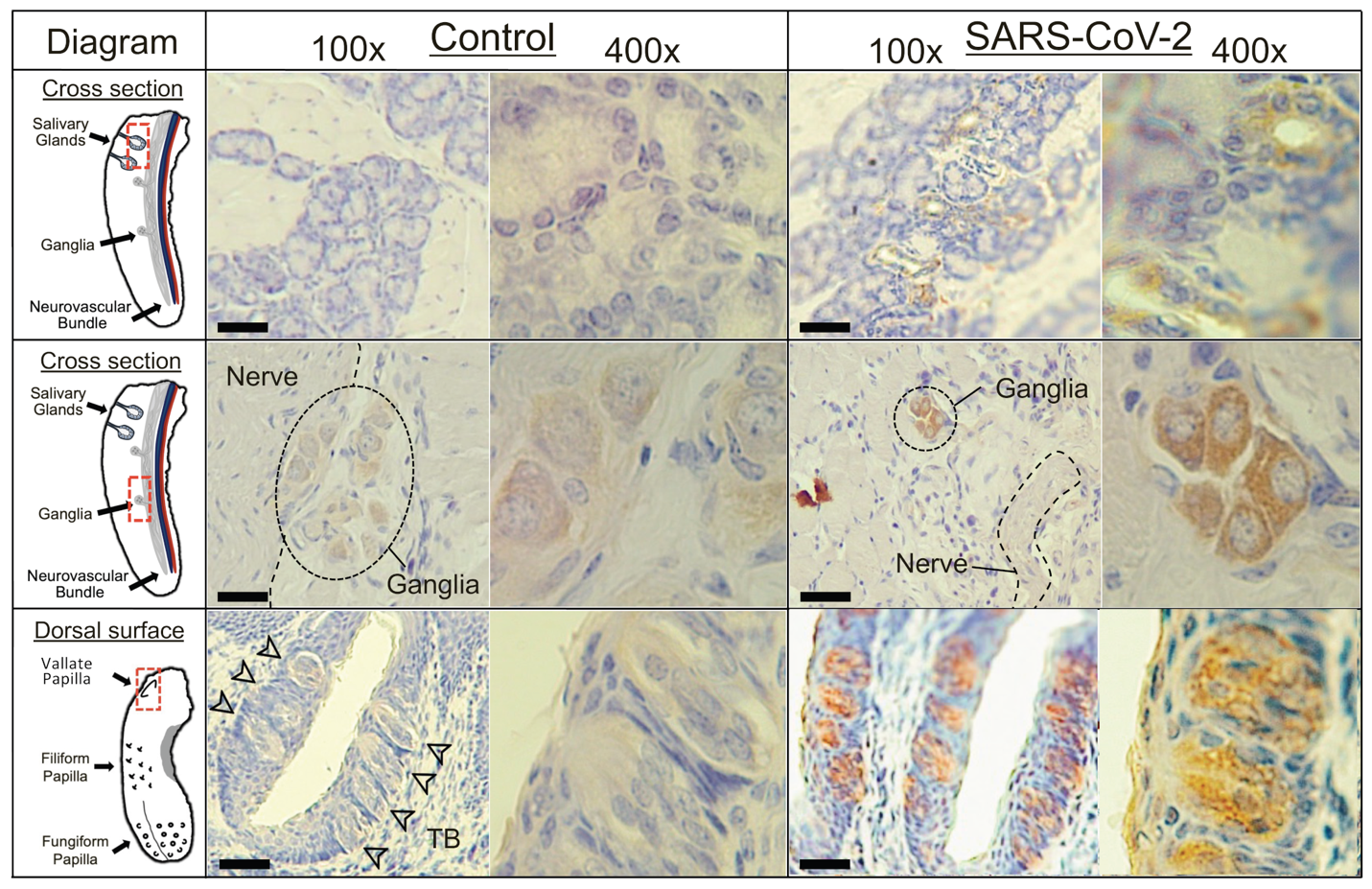
Supplemental Figure 2: SARS-CoV-2 antigen staining, 1:200 antibody dilution, immunohistochemistry experiment 1**. The tongue thin sections were labeled for SARS-CoV-2 antigen and counter-stained with hematoxylin. Representative images are presented to further illustrate the relationship between histology images and tongue anatomy and further compare positive and negative staining. Left column: Diagram of structures within the tongue, with red dashed rectangles indicating location of associated immunohistochemistry images. Middle column: control groups. Right column: infected groups. Upper row: salivary glands. Middle row: ganglia. Lower row: vallate papillae. Autonomic ganglia that are adjacent to nerves and which innervate salivary glands in the posterior tongue were labeled positively for SARS-CoV-2 antigen as a result of infection. Taste buds (TB) of vallate papillae located within the grooves of the papillae were also positive for SARS-CoV-2 antigen as a result of infection (arrowheads). Serous salivary glands, which release saliva to dissolve food molecules, were also positively labeled. Scale bars indicate 100 μm.

**
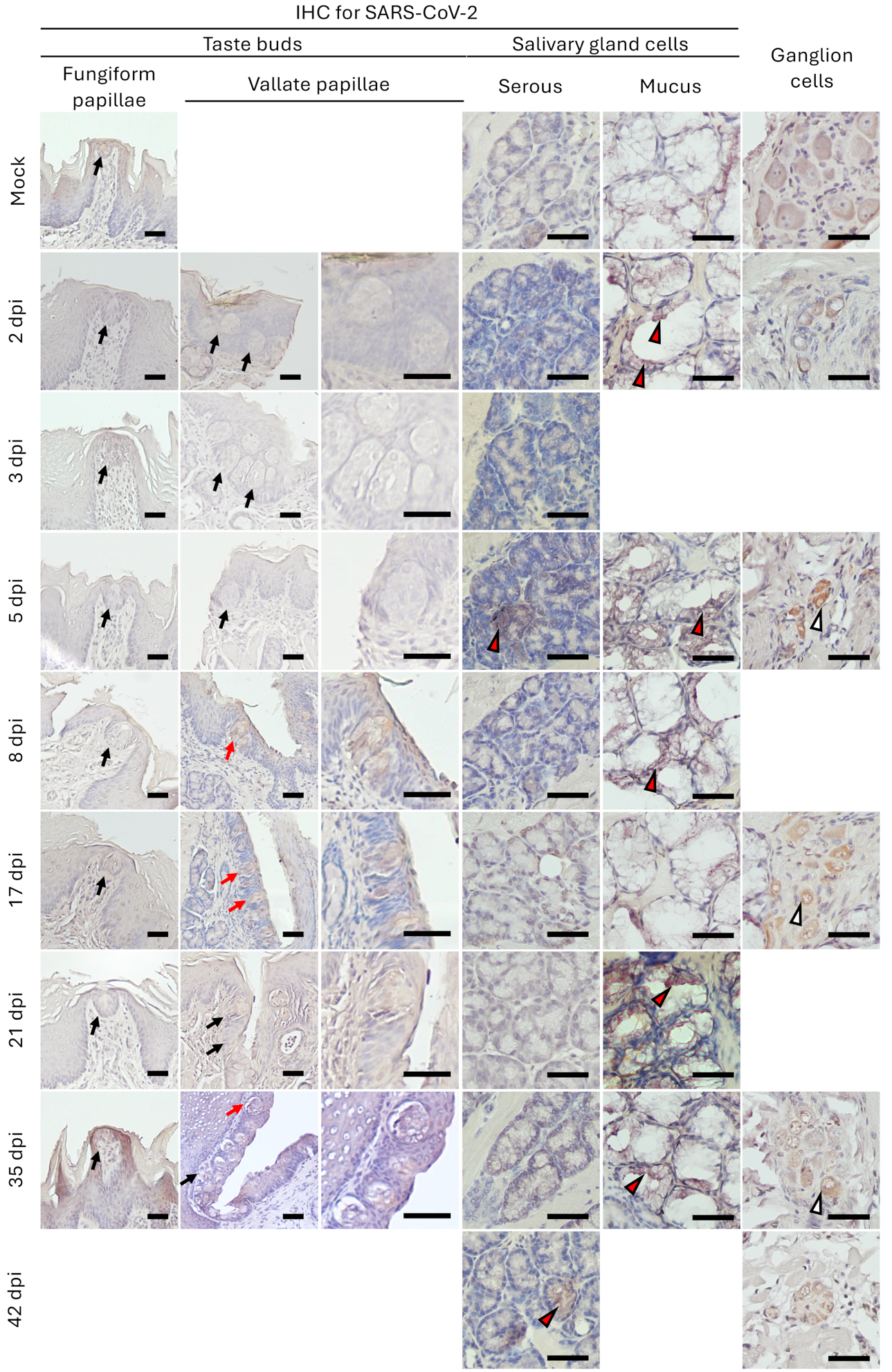
**

**Supplementary Figure 3: SARS-CoV-2 antibody staining, 1:1,000 antibody dilution, immunohistochemistry experiment 2.** The tongue thin sections were stained with SARS-CoV-2 antibodies. Representative images are presented. Columns are organized by structure. Rows are organized by days post infection (dpi) or Mock group. Black arrows indicate taste buds which scored a 0 or 1 upon grading. Red arrows indicate taste buds which scored a 2 upon grading. Red arrowheads indicate salivary glands which scored a 2 upon grading. White arrowheads indicate autonomic ganglia which scored a 2 upon grading. Scale bars indicate 100 μm.

**
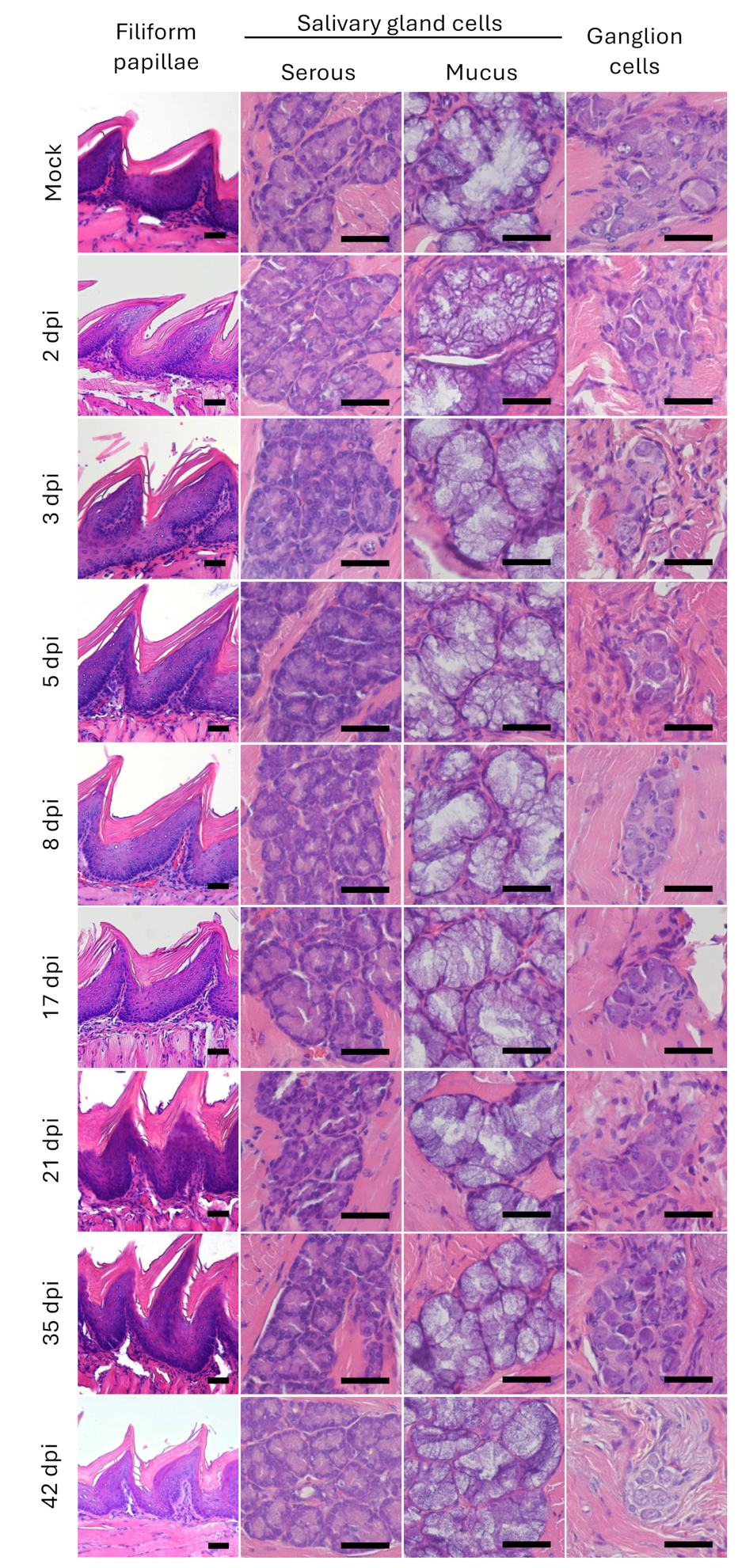
**

**Supplementary Figure 4: Hematoxylin and eosin staining.** The tongue thin sections were stained with hematoxylin and eosin. Representative images are presented. Columns are organized by structure. Rows are organized by days post infection (dpi) or Mock group. No notable de-epitheliazation or cellular infiltrates were noted within any of the structures at any time point. Scale bars indicate 100 μm.

**
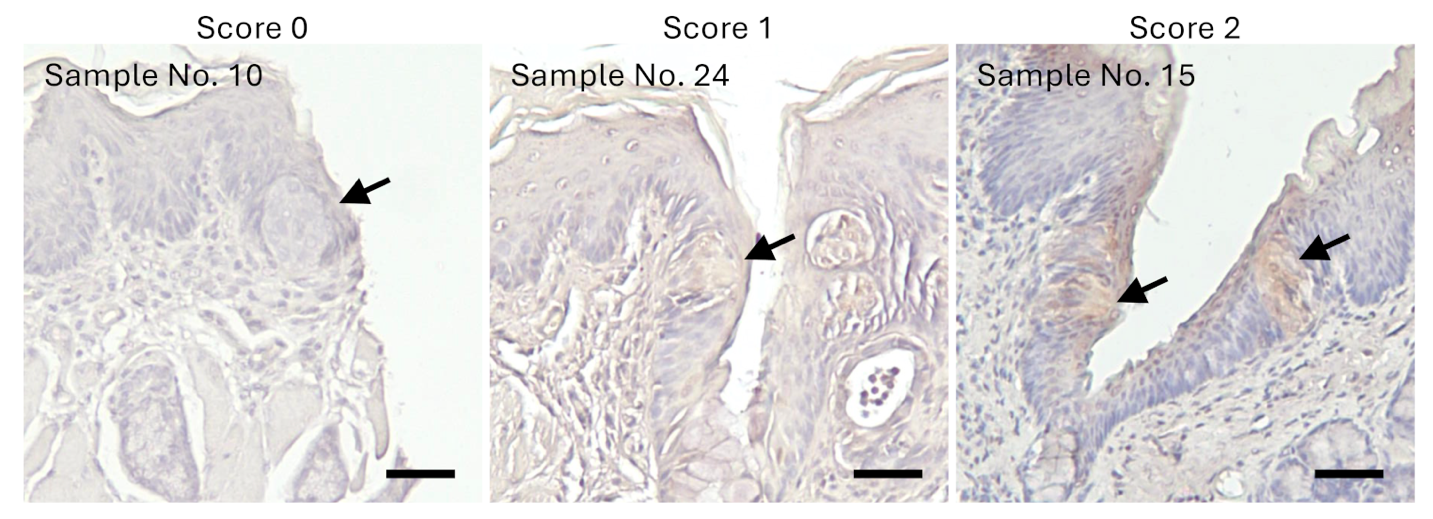
Supplementary Figure 5: SARS-CoV-2 grading example.** Structures of the tongue were graded on a scale of 0-2 for the intensity of SARS-CoV-2 labeling. A score of 0 indicates no staining, 1 indicates mild staining, and 2 indicates strong staining. This figure provides an example of the grading of vallate papillae taste buds (black arrows). Scale bars indicate 100 μm.
